# Hepatic Focal Lesion Suspicious for Hepatocellular Carcinoma in a Patient with a History of Post-Traumatic Splenectomy: The Challenge of Differential Diagnosis with Intrahepatic Splenosis—Literature Review and Case Report

**DOI:** 10.3390/diagnostics15192442

**Published:** 2025-09-25

**Authors:** Andrea Lanzafame, Giulio Perrone, Andrea Campisi, Francesco Razionale, Elena Panettieri, Enza Genco, Maria Cristina Giustiniani, Alessandro Coppola, Felice Giuliante, Francesco Ardito

**Affiliations:** 1 Hepatobiliary Surgery Unit, Foundation Policlinico Universitario A. Gemelli, IRCCS, 00168 Rome, Italy; andrea.lanzafame01@icatt.it (A.L.); drperronegiulio@gmail.com (G.P.); andrea.campisi03@icatt.it (A.C.); francesco.razionale@guest.policlinicogemelli.it (F.R.); elena.panettieri@unicatt.it (E.P.); felice.giuliante@unicatt.it (F.G.); 2 Radiology Unit, Foundation Policlinico Universitario A. Gemelli, IRCCS, 00168 Rome, Italy; enza.genco@policlinicogemelli.it; 3 Department of Pathology, Foundation Policlinico Universitario A. Gemelli, IRCCS, 00168 Rome, Italy; mariacristina.giustiniani@policlinicogemelli.it; 4 Department of Surgery, Sapienza University of Rome, 00161 Rome, Italy; 5 Department of Translational Medicine and Surgery, Università Cattolica del Sacro Cuore, 00168 Rome, Italy

**Keywords:** hepatic splenosis, hepatocellular carcinoma, differential diagnosis, splenectomy, liver resection, biopsy, pathology

## Abstract

**Background:** Hepatic splenosis (HS) is a rare para-physiological condition resulting from the ectopic implantation of splenic tissue, most commonly following traumatic or surgical splenectomy. Its radiological features can mimic those of hepatocellular carcinoma (HCC), potentially leading to misdiagnosis and unnecessary invasive procedures, such as biopsies or liver resection. **Methods:** A literature review was conducted using the PubMed database to identify all reported cases of HS. **Case Presentation**: We report the case of a 52-year-old male with an incidental finding of a liver lesion in segment V, initially suspected to be HCC, and a history of post-traumatic splenectomy. The patient had no history of underlying liver disease. Due to the lesion’s superficial location, a biopsy was not performed because of the risk of tumor rupture with subsequent bleeding or peritoneal seeding. Consequently, the patient underwent upfront laparoscopic anatomic segmentectomy of segment V. Final pathology revealed a diagnosis of intrahepatic splenosis. **Conclusions**: HS should be considered in the differential diagnosis of liver lesions in patients with a history of splenectomy but no underlying liver disease, particularly when imaging shows features suggestive of HCC, such as arterial phase hyperenhancement and portal venous washout. Awareness of this entity may prevent unnecessary invasive interventions and guide appropriate patient management.

## Simple Summary

Hepatic splenosis (HS) is a rare benign condition in which fragments of splenic tissue implant in the liver, typically following splenic trauma or splenectomy. Most patients are asymptomatic, and the condition is often incidentally detected. Radiologically, HS can closely mimic hepatocellular carcinoma (HCC), making preoperative diagnosis challenging, particularly in patients without classical HCC risk factors. Tc-99m heat-damaged red blood cell scintigraphy is a useful non-invasive diagnostic tool but is rarely performed. As a result, most cases are diagnosed through liver biopsy or surgical resection. Awareness of HS and careful consideration of patient history can prevent unnecessary interventions and guide appropriate management, highlighting the importance of including HS in the differential diagnosis of hepatic nodules.

## 1. Introduction

Hepatocellular carcinoma (HCC) is the most common primary malignant tumor of the liver. Its diagnosis is primarily based on imaging, according to the Liver Imaging Reporting and Data System (LI-RADS) criteria, which typically include arterial phase hyperenhancement (wash-in) and portal or delayed phase washout [1].

However, certain benign lesions—such as hepatic splenosis (HS)—can exhibit similar radiological features. Splenosis is a rare benign condition that typically occurs following traumatic rupture or surgical removal of the spleen, leading to the ectopic implantation of splenic tissue. Common implantation sites include the serosal surfaces of the small or large intestine, the greater omentum, the peritoneum, and, more rarely, the liver [2,3,4]. HS is usually asymptomatic and often discovered incidentally.

Differentiating HS from malignant liver lesions can be challenging, particularly in the absence of a clear medical history or underlying liver disease. Consequently, HS is often diagnosed only after liver resection for a hepatic nodule suspected to be a primary liver tumor, such as HCC.

We conducted a comprehensive literature review on HS cases presenting as HCC, with the aim of outlining the current evidence, highlighting diagnostic challenges, and summarizing therapeutic approaches. Building on this background, we describe the case of a patient with HS initially mimicking HCC, who subsequently underwent laparoscopic liver resection at our Unit [5].

The aim of this paper is to contribute to the limited body of literature on this rare condition by integrating an illustrative case with a focused review, in order to provide clinicians with practical insights for diagnosis and management.

## 2. Materials and Methods

A PubMed database search of articles published up to February 2025 has been carried out. Different combinations of the following terms have been used: hepatic splenosis; hepatocellular carcinoma; splenectomy; liver resection. Only articles published in English with available full text have been considered without limitation concerning article types (original articles, review, etc.). References reported in the selected papers have also been considered as other bibliographic sources.

Year of study, age, sex, previous splenectomy, alpha-fetoprotein level, initial diagnosis, performance of Tc-99m heat-damaged red blood cell (RBCs) scintigraphy, method of confirming diagnosis and final diagnosis were extracted from the articles.

## 3. Results

The literature review conducted using the PubMed database identified 77 articles with a total of 80 reported cases of HS between January 1993 and February 2025 which were included in the analyses (Table 1).

In 68 cases (85%), patients were male. The median age of the patients included was 51 years, with a range of 21 to 73 years, and a reported mean age of 50.0 ± 11.1 years.

With the exception of three cases in which this information was specified [29,40,64] and one case where it was not reported [77], all patients had a prior history of splenectomy. Serum alpha-fetoprotein (AFP) levels were within the normal range in 31 patients; however, it should be noted that in 40 cases, preoperative AFP values were not reported in the original publications. A preoperative diagnosis of HCC, either as a sole hypothesis or in association with other etiologies, was documented in 48.7% of cases (39 patients). The other two most frequent preoperative diagnoses were adenoma and metastasis, with seven cases each.

In the majority of cases, liver resection provided the final pathological diagnosis (46 patients, 57.5%), while liver biopsy was used in 17 patients (21.2%).

Only 15 patients (18.75%) underwent Tc-99m heat-damaged RBCs scintigraphy. Interestingly, among the subgroup of patients who underwent Tc-99m heat-damaged RBCs scintigraphy, only three subsequently received invasive diagnostic procedures: one biopsy, one liver resection, and a liver transplantation performed for an unrelated indication.

Given the rarity of this condition and the consequent diagnostic challenges, we considered of particular relevance to report a case treated at our institution that is a high-volume university Hepato-pancreato-biliary surgery center. To date, this is the only case encountered at our center, and it is presented in detail below.

### Case Presentation

**Figure 1:**
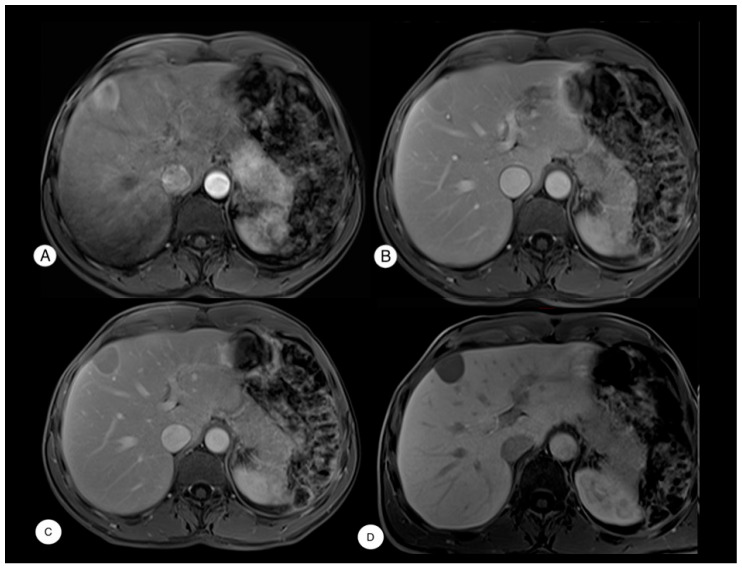
MRI of a focal lesion in hepatic segment V. T1-weighted images after contrast medium administration show that the lesion is hypervascular during the arterial phase (**A**), with an initial mild washout in the portal venous phase (**B**), becoming more pronounced in the delayed phase, where a peripheral pseudocapsule is observed (**C**). In the T1-weighted hepatobiliary phase, the lesion does not concentrate the contrast agent, appearing hypointens (**D**).

We present the clinical case of a 52-year-old male who was asymptomatic for abdominal pain, jaundice, and fever, with an incidental finding of a focal liver lesion in segment V on abdominal ultrasound, measuring approximately 3 cm. A subsequent contrast-enhanced abdominal magnetic resonance imaging (MRI) revealed a mildly enlarged liver and a superficial lesion in segment V measuring 34 × 27 × 24 mm, with regular margins. The lesion exhibited early arterial phase enhancement and rapid washout in the portal phase, along with a thin hypervascular rim—features highly suggestive of HCC according to LI-RADS criteria [1] (Figure 1). No other abdominal abnormalities were noted, except for the absence of the spleen.

Preoperative laboratory investigations were within normal limits. Tumor markers were negative: AFP 1.07 ng/mL, carcinoembryonic antigen (CEA) 1.29 ng/mL, and carbohydrate antigen 19-9 (CA19-9) 4.65 U/mL. Serology for hepatitis B virus (HBV) and hepatitis C virus (HCV) was negative. The patient had no history of alcohol abuse and no clinical or radiological evidence of liver cirrhosis. The medical history revealed that the patient had undergone an emergency splenectomy at the age of 5 following abdominal trauma. He was not taking any medications at home.

**Figure 2:**
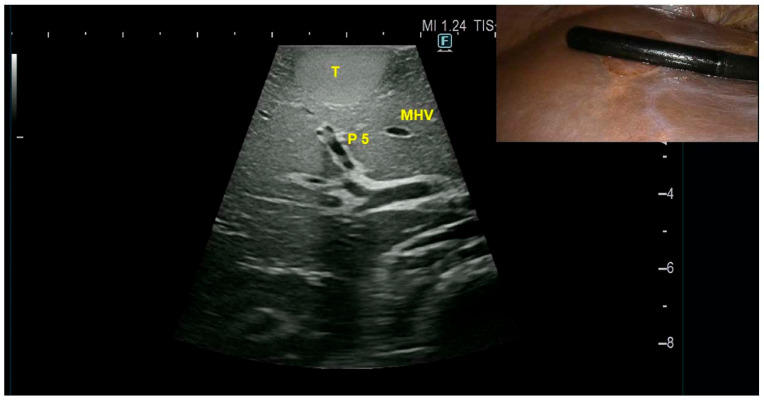
IOUS of the hepatic nodule in segment V (T) which appears hyperechoic. The tumor-bearing portal branch of segment 5 (P 5) is detected. MHV: middle hepatic vein.

The case was discussed during a multidisciplinary team meeting at our center, where the lesion was considered suspicious for HCC. Due to the superficial location of the nodule, a percutaneous biopsy was not performed because of the risk of tumor rupture, with potential bleeding or peritoneal seeding. Given the radiological suspicion of malignancy and the patient’s young age, upfront surgical management was planned, consisting of a laparoscopic anatomic segmentectomy V. The surgical technique employed in our unit has been previously described [5]. Intraoperative ultrasound (IOUS) confirmed the presence of a superficial lesion in segment V. The nodule appeared encapsulated, hyperechoic, and measured approximately 3 cm (Figure 2). The tumor-bearing portal branch of segment V was detected by IOUS (Figure 2).

**Figure 3:**
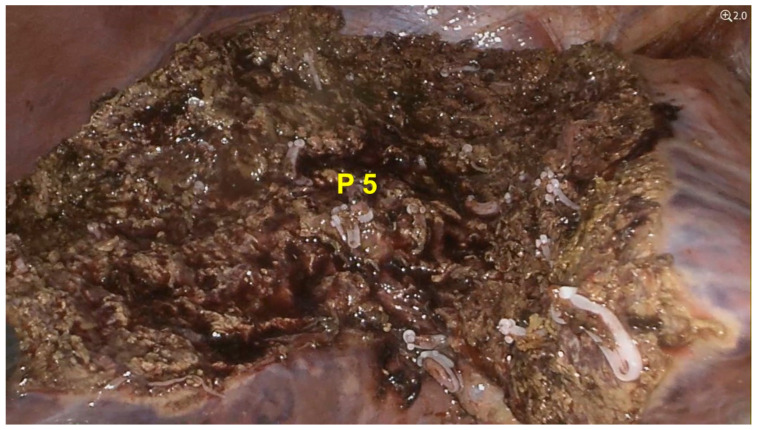
Operative field at the end of laparoscopic anatomic segmentectomy V. Stump of the Glissonean pedicle of segment V (P 5).

After clamping the tumor-bearing portal branch of segment V, indocyanine green was administered intravenously. In this way, anatomical resection was then performed using the negative staining technique. The total operative time was 4 h, with no intraoperative blood transfusion and an estimated blood loss of approximately 150 mL (Figure 3).

**Figure 4:**
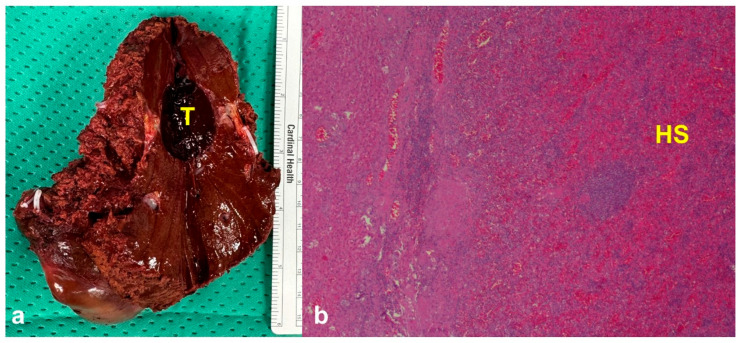
Surgical specimen of segmentectomy V showing the nodule (T) (**a**). Histological examination of the lesion revealed splenic parenchyma composed of white pulp and hyperplastic red pulp, consistent with a diagnosis of hepatic splenosis (HS) (**b**).

The postoperative course was uneventful, and the patient was discharged on postoperative day 4. Final pathology revealed ectopic splenic tissue composed of hyperplastic red and white pulp, with no evidence of neoplasia. The surrounding liver parenchyma exhibited preserved architecture, with minimal steatosis and mild fibrosis (Figure 4).

## 4. Discussion

HS is a rare benign condition characterized by the autotransplantation of splenic tissue fragments into the liver parenchyma, most commonly occurring after traumatic splenic rupture or surgical splenectomy. Once detached, splenic fragments can survive and revascularize within ectopic sites, integrating into the surrounding tissue and occasionally maintaining functional splenic activity. Although the liver represents one of the most frequent locations, ectopic implants have also been documented in other intra-abdominal regions, including the small and large intestine, the greater omentum, the mesentery, and the peritoneal surface. The reported incidence of splenosis ranges from 26% to 65% among patients with a history of traumatic splenic rupture, suggesting that the phenomenon is not uncommon but often underdiagnosed, given its typically asymptomatic course and the challenges of distinguishing it radiologically from other focal lesions.

The mechanisms underlying the ectopic implantation of splenic tissue in the liver remain incompletely understood, and several hypotheses have been proposed. The most widely accepted theory suggests that, following trauma or splenectomy, splenic tissue fragments adhere to the liver capsule and subsequently infiltrate deeper hepatic layers. Once engrafted, these fragments may survive and proliferate by establishing a vascular supply from the surrounding hepatic microcirculation, ultimately forming nodular lesions that can persist for years. In our case, the nodule was located superficially on the liver surface, a finding consistent with direct implantation of splenic tissue fragments after splenic rupture.

An alternative explanation described in the literature is the hematogenous dissemination theory, which proposes that splenic elements enter the portal venous system and reach the liver through circulation. According to this hypothesis, hypoxic stimuli within the hepatic parenchyma could promote engraftment and survival of splenic tissue, leading to ectopic splenic nodules.

Given these potential mechanisms, it is not surprising that a prior history of splenectomy or abdominal trauma is frequently reported in patients diagnosed with hepatic splenosis. In fact, our review of the literature confirms this strong association, showing that the vast majority of patients (*n* = 76; 95.0%) had previously undergone splenectomy. This clinical correlation underscores the importance of a thorough surgical history when evaluating hepatic nodules suspicious for HS.

Ectopic splenic tissue may retain functional activity, contributing to splenic immunologic function, or may remain inactive; regardless, it is universally considered a benign entity. HS is typically asymptomatic and often discovered incidentally during imaging or surgery. In rare cases, however, clinical manifestations can occur depending on the location and size of the ectopic tissue, including gastrointestinal bleeding, abdominal pain, appendicitis, intestinal obstruction, or torsion of the splenic implant. Such complications may necessitate surgical intervention to relieve symptoms or prevent further morbidity [3,4].

Our literature review confirms a clear male predominance among reported cases of HS, with the highest incidence occurring between the fourth and fifth decades of life. These findings highlight that HS most commonly affects middle-aged adults, reflecting both the typical age at which splenectomy or traumatic splenic injury occurs and the long latency period over which ectopic splenic tissue can persist and become clinically detectable.

The primary challenge in diagnosing HS lies in differentiating it from malignant liver lesions, such as hepatocellular carcinoma or metastatic disease. Radiological characteristics of HS often closely mimic those of HCC, including arterial phase hyperenhancement, delayed washout during the portal venous phase, and hypointensity on hepatobiliary phase imaging. The diagnostic difficulty is further compounded in the absence of classical risk factors for HCC, such as chronic hepatitis B or C infection, heavy alcohol use, or underlying cirrhosis.

**Figure 5:**
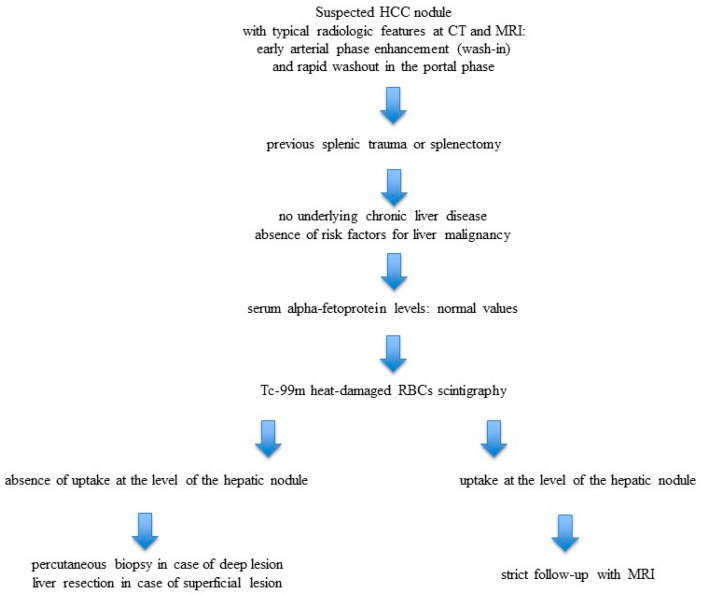
Describes a possible diagnostic algorithm in case of suspicious HS.

Our review of the literature demonstrates that a preoperative suspicion of HS was rarely considered, occurring in only 20% of cases. In contrast, the majority of patients (67.5%; 54 patients) were initially suspected of having a malignant tumor. Among these, the most frequent preoperative diagnosis was HCC, accounting for 48.7% of cases (39 patients). These findings underscore the tendency of HS to present with radiological features that closely resemble HCC, often leading to misdiagnosis and, in some cases, unnecessary invasive procedures. The results highlight the importance of including HS in the differential diagnosis of hepatic nodules, particularly in patients with a history of splenectomy or abdominal trauma, to avoid overtreatment and optimize patient management. We report a possible diagnostic algorithm in case of suspicious HS (Figure 5)

Tc-99m heat-damaged RBCs scintigraphy represents a reliable, non-invasive technique for confirming the diagnosis of splenosis [81,82]. However, despite its diagnostic utility, this imaging modality was infrequently employed in the reported cases, being performed in only 15 patients (18.75%). One likely explanation for this low utilization is its limited availability in many clinical centers. Moreover, it is important to recognize that the diagnostic sensitivity of this technique may be affected by technical factors. Improper preparation of heat-damaged RBCs—such as insufficient or excessive heating—can result in false-negative findings, potentially leading to missed diagnoses [4]. As reported in Table 1, in two cases, in addition to scintigraphy, one patient underwent biopsy and another hepatic resection. Notably, the biopsy was performed before scintigraphy, which subsequently confirmed the diagnosis of splenosis [16]. In Kawada et al. [68], the authors noted that due to the rarity of splenosis, they opted for hepatic resection for definitive histological confirmation despite positive scintigraphy.

The majority of patients, therefore, underwent liver resection, with 46 cases (57.5%) treated surgically. Notably, this trend has not decreased over the past five years; among 14 recently reported cases, 10 patients (71.4%) underwent hepatic resection. These data underscore the persistent challenges in achieving a definitive preoperative differential diagnosis between HS and malignant hepatic lesions, highlighting the ongoing reliance on surgical intervention for diagnostic confirmation.

## 5. Conclusions

In conclusion, consistent with the current literature, HS should be considered in the differential diagnosis of patients presenting with one or more hepatic nodules that mimic HCC on contrast-enhanced imaging studies. This is particularly important in patients without underlying chronic liver disease but with a history of splenectomy or abdominal trauma. Among the available diagnostic modalities, Tc-99m heat-damaged RBC scintigraphy represents the most accurate non-invasive option, as it can confirm splenic tissue by demonstrating selective uptake of labeled erythrocytes. Typical findings include focal radiotracer accumulation within the hepatic nodules, which is highly specific and allows for a confident diagnosis, thereby avoiding unnecessary invasive procedures such as biopsy or liver resection. Increased awareness among clinicians and radiologists is therefore crucial to improve preoperative diagnostic accuracy, minimize overtreatment, and optimize patient management.

## Figures and Tables

**Table 1:** Results from the literature review: 80 reported cases of HS between January 1993 and February 2025.

Author (Year)	Age/Sex	Previous Splenectomy	Alpha-Fetoprotein (ng/mL)	Primary Diagnosis	99mTc Scintigraphy	Treatment	Final Diagnosis
Yoshimitsu K (1993) [6]	51/F	Yes	Normal	HCC	No	Surgery	HS
Gruen DR (1997) [7]	38/F	Yes	N/A	Adenoma vs. FNH	No	Surgery	HS
Davidson LA (1997) [8]	54/M	Yes	N/A	N/A	No	Necropsy	HS
D’ Angelica M (1998) [9]	38/F	Yes	N/A	Adenoma vs. FNH	No	Surgery	HS
Foroudi F (1999) [10]	59/M	Yes	N/A	Metastasis	Yes	-	HS
De Vuysere S (2000) [11]	50/M	Yes	Normal	HS	No	Biopsy	HS
Pekkafali Z (2002) [12]	21/M	Yes	N/A	HS	Yes	-	HS
Gamulin A (2002) [13]	49/M	Yes	N/A	Lymphoma	No	Surgery	HS
Lee JB (2002) [14]	43/M	Yes	Normal	HCC	No	Surgery	HS
Kim KA (2003) [15]	43/M	Yes	Normal	HCC	No	Surgery	HS
Di Costanzo GG (2004) [16]	58/M	Yes	412	HCC	Yes	Biopsy	HS
Di Costanzo GG (2004) [16]	48/F	Yes	327	HCC	No	Biopsy	HS
Kondo M (2004) [17]	55/M	Yes	N/A	HCC	No	Biopsy	HS
Ferraioli G (2006) [18]	40/M	Yes	Normal	HS	No	Biopsy	HS
Yeh ML (2008) [19]	64/M	Yes	Normal	HCC	No	Surgery	HS
Lu HC (2008) [20]	59/M	Yes	Normal	HS	Yes	-	HS
Choi GH (2008) [21]	32/M	Yes	17.3	HCC	No	Surgery	HS
Grande M (2008) [22]	41/M	Yes	Normal	HS	Yes	-	HS
Imbriaco M (2008) [23]	39/M	Yes	N/A	Hepatic Tumor	No	Surgery	HS
Nakajima T (2008) [24]	41/M	Yes	N/A	HS	No	Biopsy	HS
Yu H (2009) [25]	54/M	Yes	Normal	Hepatoma	No	Surgery	HS
Abu Hilal M (2009) [26]	60/M	Yes	Mild rise	HCC	No	Surgery	HS
Kashgari AA (2009) [27]	52/M	Yes	Normal	HCC	No	Biopsy	HS
Menth M (2009) [28]	43/M	Yes	6.4	HCC	Yes	-	HS
Mescoli C (2010) [29]	68/F	No	N/A	FNH	No	Biopsy	HS
Mescoli C (2010) [29]	54/M	Yes	N/A	Metastasis	No	Surgery	HS
Tsitouridis I (2010) [30]	63/M	Yes	N/A	HS	No	Surgery	HS
Tsitouridis I (2010) [30]	64/M	Yes	N/A	Peritoneal implant	No	Biopsy	HS
Kang KC (2011) [31]	54/M	Yes	N/A	Metastasis	No	Surgery	HS
Liu Y (2012) [32]	49/F	Yes	N/A	Metastasis	No	Surgery	HS
Li H (2012) [33]	61/M	Yes	N/A	HS	No	Biopsy	HS
Liu K (2012) [34]	38/M	Yes	Normal	Hepatic Tumor	No	Surgery	HS
Inchingolo R (2013) [35]	53/M	Yes	Normal	HCC vs. Adenoma	No	Surgery	HS
Krawczyk M (2013) [36]	39/F	Yes	N/A	HS	Yes	-	HS
Röther M (2013) [37]	62/M	Yes	Normal	HCC	No	Surgery	HS
Leong CW (2013) [38]	56/M	Yes	N/A	Carcinoid NET	No	Surgery	HS
Kandil TS (2014) [39]	45/F	Yes	Normal	HCC	No	Surgery	HS
Sato N (2014) [40]	58/M	No	Elevated	HCC	No	Surgery	HS
Tinoco Gonzales J, (2014) [41]	60/M	Yes	N/A	HCC	No	Surgery	HS
Wu C (2015) [42]	33/M	Yes	Normal	HCC	No	Surgery	HS
Liu C (2015) [43]	33/M	Yes	Normal	HCC	No	Biopsy	HS
Li T (2015) [44]	67/F	Yes	Elevated	HCC	No	Surgery	HS
Tamm A (2015) [45]	43/M	Yes	N/A	Suspicious nodule	Yes	-	HS
Grambow E (2015) [46]	53/M	Yes	Normal	HCC	No	Surgery	HS
Toktas O (2015) [47]	40/F	Yes	N/A	Suspicious nodule	No	Surgery	HS
Fung A (2016) [48]	55/M	Yes	Normal	Hepatic Tumor	No	Surgery	HS
He ZL (2016) [49]	51/M	Yes	N/A	Metastasis	No	Biopsy	HS
Jereb S (2016) [50]	22/M	Yes	Normal	Metastasis	No	Biopsy	HS
De Riggi MA (2016) [51]	31/M	Yes	N/A	Hepatic Tumor	No	Surgery	HS
Wang MY (2017) [52]	42/M	Yes	1.531	HCC	No	Surgery	HS
Keck C (2017) [53]	66/M	Yes	Normal	HS	No	Biopsy	HS
Wang WC (2017) [54]	54/M	Yes	Normal	HCC	No	Surgery	HS
Somsap K (2017) [55]	51/M	Yes	Normal	HCC	No	Surgery	HS
Teles GNS (2018) [2]	73/M	Yes	Normal	Hepatic Tumor	No	Surgery	HS
Aramoana J (2018) [56]	58/M	Yes	N/A	HS	No	Surgery	HS
Vergara D (2018) [57]	69/M	Yes	Normal	HS	No	Biopsy	HS
Guzman Rojas P (2018) [58]	43/M	Yes	N/A	Adenoma	No	Biopsy	HS
Budak E (2018) [59]	46/M	Yes	N/A	HCC vs. HS	Yes	-	HS
Varghese J (2018) [60]	50/M	Yes	N/A	Suspicious nodule	No	CT splenic-like enhancement	HS
Xuan Z (2018) [61]	54/M	Yes	Normal	HCC	No	Surgery	HS
Smolen B (2019) [62]	35/M	Yes	Normal	Adenoma vs. FNH	Yes	-	HS
Guedes TP (2019) [63]	68/M	Yes	Normal	HCC vs. adenoma	No	Surgery	HS
Ananthan K (2019) [64]	57/M	No	Normal	HS	Yes	-	HS
Kosydar SR (2019) [65]	38/M	Yes	N/A	HS	No	-	HS
Luo X (2019) [3]	41/M	Yes	Normal	Metastasis	No	Surgery	HS
Martín-Marcuartu JJ, (2020) [66]	66/M	Yes	N/A	HS	Yes	Liver transplantation for other reason	HS
Sansone V (2020) [67]	46/M	Yes	N/A	Adenoma vs. Hemangioma	No	Surgery	HS
Kawada S (2020) [68]	39/M	Yes	N/A	Suspicious nodule	Yes	Surgery	HS
Zhong X (2021) [69]	55/M	Yes	N/A	Suspicious nodule	No	Surgery	HS
Richardson L (2021) [70]	68/M	Yes	N/A	HS	No	-	HS
Djekidel M (2021) [71]	29/F	Yes	N/A	Hepatic Tumor	Yes	-	HS
Anderson M (2022) [72]	54/M	Yes	N/A	Hepatic Tumor	Yes	-	HS
Pessarelli T (2022) [73]	56/M	Yes	N/A	HCC	No	Surgery	HS
M S P (2023) [74]	57/M	Yes	N/A	Suspicious nodule	No	Surgery	HS
Umeda I (2023) [75]	46/M	Yes	Normal	HCC	No	Surgery	HS
Rosi M (2024) [76]	50/M	Yes	N/A	HCC	No	Surgery	HS
Hu ZY (2024) [77]	53/M	N/A	N/A	Hepatic Tumor	No	Surgery	HS
Ikeda N (2024) [78]	56/F	Yes	Normal	HCC	No	Surgery	HS
Escribano Cruz S (2024) [79]	47/M	Yes	N/A	Adenoma	No	Biopsy	HS
Marrana F (2025) [80]	51/M	Yes	N/A	Adenoma	No	Surgery	HS

## Data Availability

The original contributions presented in this study are included in the article. Further inquiries can be directed to the corresponding authors.

## Abbreviations

The following abbreviations are used in this manuscript:

AFP	Alpha-fetoprotein
CEA	Carcinoembryonic antigen
CA19-9	Carbohydrate antigen 19-9
HBV	Hepatitis B virus
HCV	Hepatitis C virus
HCC	Hepatocellular carcinoma
HS	Hepatic splenosis
IOUS	Intraoperative ultrasound
LI-RADS	Liver Imaging Reporting and Data System
MHV	Middle hepatic vein
MRI	Magnetic resonance imaging
